# Temperature variation makes ectotherms more sensitive to climate change

**DOI:** 10.1111/gcb.12240

**Published:** 2013-05-29

**Authors:** Krijn P Paaijmans, Rebecca L Heinig, Rebecca A Seliga, Justine I Blanford, Simon Blanford, Courtney C Murdock, Matthew B Thomas

**Affiliations:** * Center for Infectious Disease Dynamics and Department of Entomology, The Pennsylvania State UniversityUniversity Park, PA, 16802, USA; † Barcelona Centre for International Health Research (CRESIB, Hospital Clínic-Universitat de Barcelona)Carrer Roselló 132, 4th floor, Barcelona, E-08036, Spain; ‡ GeoVISTA Center Department of Geography, The Pennsylvania State UniversityUniversity Park, PA, 16802, USA; § Department of Biology, The Pennsylvania State UniversityUniversity Park, PA, 16802, USA

**Keywords:** *Anopheles stephensi*, climate change, conservation, diurnal temperature fluctuation, ectotherm fitness, Jensen's inequality, thermal fitness curve, thermal reaction norm

## Abstract

Ectotherms are considered to be particularly vulnerable to climate warming. Descriptions of habitat temperatures and predicted changes in climate usually consider mean monthly, seasonal or annual conditions. Ectotherms, however, do not simply experience mean conditions, but are exposed to daily fluctuations in habitat temperatures. Here, we highlight how temperature fluctuation can generate ‘realized’ thermal reaction (fitness) norms that differ from the ‘fundamental’ norms derived under standard constant temperatures. Using a mosquito as a model organism, we find that temperature fluctuation reduces rate processes such as development under warm conditions, increases processes under cool conditions, and reduces both the optimum and the critical maximum temperature. Generalizing these effects for a range of terrestrial insects reveals that prevailing daily fluctuations in temperature should alter the sensitivity of species to climate warming by reducing ‘thermal safety margins’. Such effects of daily temperature dynamics have generally been ignored in the climate change literature.

## Introduction

**Figure 1:**
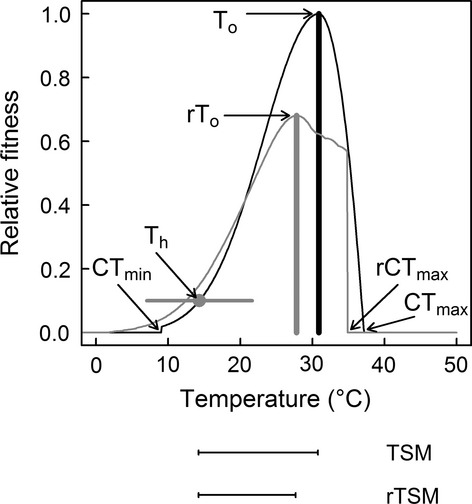
Effect of daily temperature variation on ectotherm fitness. Fundamental performance curve (relative fitness; black line) under constant temperature conditions and realized performance when appropriate daily temperature variation is considered (grey line) for the temperate terrestrial insect *Muscidifurax zaraptor*. Gray data point and horizontal grey line represent the mean habitat temperature (*T*_h_) and the average habitat temperature range, respectively. CT_min_, critical minimum temperature; (r)*T*_o_, (realized) optimum temperature; (r)CT_max_, (realized) critical maximum temperature; and (r)TSM, (realized) thermal safety margin.

The relationship between ectotherm life-history traits and temperature is typically characterized by a nonlinear asymmetric curve, defining the optimum temperature (*T*_o_) and the operative temperature range between the critical minimum temperature (CT_min_) and the critical maximum temperature (CT_max_) (Fig. 1). These curves are often used to assess the sensitivity of ectotherm species to climate warming, sometimes considering individual life-history traits (Amarasekare & Savage, 2012), or composite fitness metrics such as the intrinsic rate of increase (Deutsch *et al*., 2008; Tewksbury *et al*., 2008). Approaches include simple use of curves to track expected changes in overall fitness (Deutsch *et al*., 2008; Tewksbury *et al*., 2008), in CT_min_ or CT_max_ (Piyaphongkul *et al*., 2012; Ribeiro *et al*., 2012) or in thermal performance breadth (Angert *et al*., 2011) as a result of changing thermal environments. In addition, studies use measures such as the Thermal Safety Margin (TSM), which defines the amount of warming possible before habitat temperatures (*T*_h_) reach and ultimately exceed the thermal optimum (*T*_o_) (Deutsch *et al*., 2008; Huey *et al*., 2009; Hoffmann, 2010; Hofmann & Todgham, 2010; Clusella-Trullas *et al*., 2011; Bonebrake & Deutsch, 2012; Krenek *et al*., 2012).

Thermal reaction norms and fitness curves tend to be derived from constant temperature experiments conducted under controlled laboratory conditions (e.g., see species listed in Deutsch *et al*., 2008). However, temperature is highly dynamic (Geerts, 2003; Paaijmans *et al*., 2010) and numerous studies provide evidence of insects and other ectotherms ‘integrating’ the effects of temperature during the daily cycle (Liu *et al*., 1995; Kingsolver *et al*., 2009; Paaijmans *et al*., 2010; Bozinovic *et al*., 2011; Duncan *et al*., 2011; Estay *et al*., 2011; Folguera *et al*., 2011). Accordingly, short-term environmental variance has the potential to affect life-history traits and fitness above and beyond the effects of mean temperatures alone (Ruel & Ayres, 1999; Martin & Huey, 2008; Folguera *et al*., 2009, 2011; Terblanche *et al*., 2010; Bozinovic *et al*., 2011; Clusella-Trullas *et al*., 2011). As climate change will not only alter mean temperatures, but also the daily temperature ranges (Easterling *et al*., 1997, 2000), understanding these effects is necessary to define the ‘realized’ thermal reaction norms (i.e., the actual fitness curves observed under variable conditions in nature) for different species and to quantify vulnerability to climate warming.

Here, using the mosquito *Anopheles stephensi* as a model organism, we ask whether the ‘fundamental’ thermal reaction norms established under constant temperature conditions differ from those derived under more natural fluctuating temperatures. We illustrate how daily temperature fluctuations can lower both the optimum and critical maximum temperatures of thermal reaction norms. We then generalize these effects for a range of terrestrial insects using a rate summation modeling approach. This analysis reveals that daily temperature fluctuations will tend to reshape the fundamental fitness curve, reducing the temperature optimum and hence, the TSM. Together, the empirical and theoretical data demonstrate that predicting the impacts of climate on ectotherm fitness requires a better understanding of the effects of short-term temperature dynamics.

## Materials and methods

### Empirical studies exploring the effects of temperature variation on life-history traits

Experiments were carried out in incubators (Percival Scientific Inc., Perry, IA, USA and Conviron, Canada, accuracy: ±0.5 °C), at 90 ± 10% relative humidity and a 12L : 12D photoperiod (L: 12:00 hours to 0:00 hours). Temperature was monitored closely with temperature loggers (OM-62; Omega, Stamford, CT, USA) at 15 min intervals. To exclude the potential effect of incubator, incubator programs were changed between experiments (e.g., an incubator running at a low but constant temperature was then programmed to fluctuate around a high mean temperature, etc.).

Immature *An. stephensi* (first instar larvae; <24 h old) were reared in plastic cups with 3 cm of distilled water (diameter 7 cm; 115 mL of water) and 50 larvae per cup (or 2.3 larvae cm^−2^). A small water volume was chosen to ensure water temperature tracked the temperature in the incubators. Live larvae and pupae were counted and cups cleaned daily. Immatures were placed back in clean water that was stored overnight at the respective temperature, to avoid temperature variation due to daily changing of the water. Larvae were fed 0.3 mg of tropical fish food (Tetrafin®) per larva per day. Emerged adult mosquitoes were counted (at the end of the night cycle) and removed. Daily development rate (reciprocal of time until adult emergence) and survival to adult were measured during different temperature experiments, with six replicate cups per temperature treatment.

Utilizing these general methods we conducted a suite of experiments to explore effects of mean temperature and daily temperature variation on specific aspects of thermal reaction norms.

#### The fundamental thermal reaction norm

Larvae were reared at constant temperatures, ranging from 16 to 36 °C, with 2 °C increments, to obtain the fundamental thermal reaction norms (and a measure of the optimum, *T*_o_, and the critical maximum temperature, CT_max_) for development and survival. This temperature range was selected on the basis of pilot data and those published for *An. gambiae* (Bayoh & Lindsay, 2003). Note that critical minimum (CT_min_) and maximum (CT_max_) temperatures are commonly derived from experiments whereby temperature is slowly decreased or increased, and some kind of short-term physiological or behavioral response is recorded (e.g., ability of an insect to right itself). In our experiments, we define CT_min_ and CT_max_ more ecologically as those temperatures at which mosquito survival through to adulthood is zero (which could still mean larvae surviving for many days but failing to emerge from pupae). This measure is not equivalent to the traditional upper or low lethal temperatures, which again tend to be defined based on mortality following single short-term exposures to temperature extremes (Piyaphongkul *et al*., 2012; Ribeiro *et al*., 2012).

#### Effects of fluctuation at different points on the reaction norm

Based on the fundamental reaction norms, we examined the effects of diurnal temperature variation on larval development and survival at 18 °C (near CT_min_), 32 °C (near *T*_o_ for development), and 26 °C (an intermediate temperature). We evaluated constant temperatures and diurnal temperature ranges (DTRs) of 8 °C (i.e., ±4) and 12 °C (i.e., ±6) around the same means, with the daily temperature profiles described by the Parton & Logan temperature model (see model details below). These temperature ranges are commonly experienced by anopheline mosquitoes (Paaijmans *et al*., 2008).

#### Effects of temperature variation on the temperature optimum

Here, we investigated the effects of a DTR of 12 °C around subtly different means of 28, 30, and 32 °C to examine more precisely whether daily fluctuations in temperature affected the optimum temperature for development and survival.

For this set of experiments, we ran a full factorial generalized linear model (GZLM) analysis to assess how mean temperature, diurnal temperature fluctuation, and replicate influence larval development time (days) and the number of emerging adult mosquitoes. We assumed a linear distribution (identity link function) and a Poisson distribution (log link function) for the GZLMs with development time and number of emerging adult mosquitoes as response variables, respectively. We assessed goodness of fit of the final models through model deviance/d.f. scores and model residuals. Reduced models were achieved by eliminating the highest order, nonsignificant interactions through backward elimination. All post hoc tests were Bonferroni corrected, and analyses were run in spss 20.0 (IBM Corporation, Armonk, NY, USA). In addition, for the temperature optima experiment, development times were transformed to meet the assumption of normality.

#### Effects of temperature variation on the critical maximum temperature

To investigate the effects of temperature variation on CT_max_, we measured survival at a range of high mean temperatures with and without different DTRs. Specifically we tested 33 and 35 °C with a DTR of 8, 31 and 33 °C with a DTR of 12, and 29.5 and 31.5 °C with a DTR of 16 °C. In addition, we ran a further assay to examine the impact of smaller fluctuations (DTRs of 3, 5, and 7 °C) around 35 °C only. Our aim in these experiments was to determine the temperature combinations at which mosquito survival through to adulthood was zero.

### Theoretical analysis of thermal safety margins under current and future climate conditions

We now extend our approach to explore the effects of temperature fluctuation on the TSM of 29 terrestrial insect species. These species represent a subset of those presented in an earlier study by Deutsch *et al*. (2008) and cover diverse taxa from a range of temperate and tropical habitats. We used rate summation (Liu *et al*., 1995) to accumulate fitness rates at hourly intervals using the ‘fundamental’ fitness curves for each species and the relevant diurnal temperature cycles for the local environment. This approach generates a ‘realized’ thermal fitness curve based on the average daily temperature variation experienced by the insect in nature. We then compare the TSMs based on the fundamental thermal fitness curve with TSMs based on the realized curves.

The asymmetric thermal curves for relative insect fitness (the intrinsic rate of increase scaled to 1 as a maximum) were calculated using a Gaussian quadratic function described by Deutsch *et al*. (2008). In this earlier study, the authors calculated the critical maximum (CT_max_) and optimum temperature (*T*_o_) for 38 terrestrial insect species using available empirical data on intrinsic growth rate at several temperatures. However, rather than calculating the critical temperature (CT_min_) from the quadratic function, the authors provided an operational definition of CT_min_. While this was appropriate for their study, for our rate summation approach we needed the full thermal fitness curves so we refitted Gaussian quadratic models to the empirical data from the original source literature. When the original fitness data were presented as a figure only, the figure was digitized using Engauge Digitizer to obtain the values. To increase the robustness of our model, we used two additional criteria: (1) there should be at least three fitness data points below *T*_o_ and (2) the relative fitness value of one of these data points had to be lower than 0.5. Based on these criteria, nine of the original 38 species were omitted from our analysis of TSM (species 2, 7, 8, 10, 15, 16, 17, 21, and 36).

The appropriate variation in habitat temperature for each species was estimated using the mean monthly minimum and maximum temperatures obtained from WorldClim, version 1.4 (release 3; http://www.worldclim.org, Hijmans *et al*., 2005). Mean monthly minimum and maximum temperature surfaces were generated from the period 1960–1990 (referred to as contemporary temperatures) using weather station records containing at least 10 years of data in this period. These surfaces were imported into ESRI^tm^ ArcGIS ArcView 9.3 and used to obtain the climatic data for the geographical locations of the 29 insect species. Daily minimum and maximum temperature data for 2080 were obtained for UKMO_HadCM3 (Hadley Centre for Climate Prediction and Research, Met Office, UK) using SRES – A2A from http://www.ccafs-climate.org/data/, version IPCC 4, at a spatial resolution of 2.5 min. This climate model and scenario is one of the several possible combinations but has been used in a number of recent ecological studies examining possible effects of climate change (Heikkinen *et al*., 2010; Lassalle *et al*., 2010; Milanovich *et al*., 2010; Jaramillo *et al*., 2011). Climate data were again imported in ArcView 9.3 and temperature information extracted for the locations of the 29 insect species, as above.

We considered only months in which individual species were expected to be active and where physiological processes such as growth and reproduction are likely to occur, i.e., mean habitat temperature >CT_min_, the critical minimum temperature for fitness, and months where the minimum temperature is greater than 0 °C, which sometimes excluded the winter months. Applying some form of seasonal constraint is consistent with other studies (e.g., Clusella-Trullas *et al*., 2011), but differs from the original approach of Deutsch *et al*. (2008), who considered year-round mean temperatures. How habitat temperature is characterized will influence the value of the TSM. However, our focus here is not on the absolute values of TSM but the relative change that results from the use of fluctuating temperatures compared with mean temperatures.

We used a standard method for generating realistic variations in the daily air temperature, whereby the phase and form of the diurnal rhythm of air temperature are given by a sinusoidal progression during daytime and a decreasing exponential curve during the night (Parton & Logan, 1981). The air temperature was modeled at 1 h intervals using the contemporary and the forecast minimum and maximum temperatures, assuming a day length of 12 h.

Combining the fundamental fitness curves for each species and the relevant diurnal temperature cycles for the local environment, fitness was calculated using either the mean habitat temperature, or using rate summation to accumulate fitness at hourly intervals. The rate summation approach generates a realized thermal fitness curve with a realized optimum temperature (r*T*_o_), based on the average daily temperature variation experienced by the insect in nature. We compared TSMs based on the fundamental fitness curves (*T*_o_–*T*_h_) with those based on the realized fitness curves (r*T*_o_–*T*_h_).

All model calculations and figures were produced in the statistical package R (R Development Core Team, 2012).

## Results

### Empirical studies

**Figure 2:**
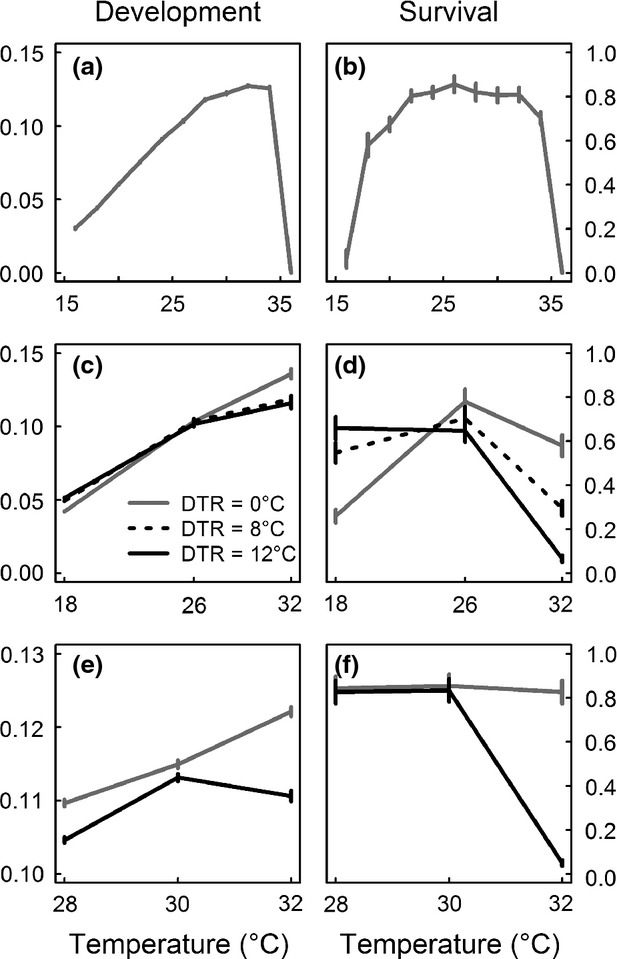
Impact of constant and variable temperatures on insect life-history traits. (a, b) Fundamental thermal reaction norm for (a) daily development rate and (b) survival of *Anopheles stephensi* mosquito immatures measured at 11 constant temperatures. (c, d) Effects of fluctuation at different points on the fundamental curve: Estimated marginal mean (c) development rate and (d) survival at mean temperatures of 18, 26, or 32 °C, combined with daily temperature ranges (DTRs) of 0, 8, or 12 °C. (e, f) Effects of temperature variation on the temperature optimum: Estimated marginal mean (e) development rate and (f) survival at mean temperatures of 28, 30 or 32 °C, combined with DTRs of 0 or 12 °C. The vertical error bars in all panels represent the standard error of the mean.

The ‘fundamental’ thermal reaction norms established under constant temperature conditions showed the operative range for mosquito larval development and survival to extend from around 15 °C (estimated CT_min_) to 36 °C (CT_max_), with an optimum temperature (for development) of around 32 °C (Fig. 2a and b). The addition of realistic daily temperature variation changed the shape of these fundamental reaction norms. Temperature variations around a cool mean temperature (18 °C) significantly increased development rate (DTR 8 °C, *P *<* *0.0001; DTR 12 °C, *P *<* *0.0001) and survival (DTR 8 °C, *P *<* *0.0001; DTR 12 °C, *P *<* *0.0001), compared with the constant baseline temperature (Fig. 2c and d). In contrast, temperature variation around a warm mean temperature (32 °C) significantly reduced development rate (DTR 8 °C, *P *<* *0.0001; DTR 12 °C, *P *<* *0.0001) and survival probability (DTR 8 °C, *P *<* *0.0001; DTR 12 °C, *P *<* *0.0001). The effects of the larger DTR were significantly greater than the smaller DTR (*P *<* *0.0001) for development and survival at fluctuation around 18 °C and for survival at fluctuation around 32 °C. At the intermediate temperature (26 °C), fluctuation had no significant effects relative to the baseline constant temperature.

Daily temperature variation also affected the estimate of the temperature optimum. In the absence of any variation, larval development rate increased as mean temperatures shifted from 28 to 30 and then to 32 °C (*P *<* *0.0001), although there was no effect on survival (Fig. 2e and f). With no evidence of a turnover, these data suggest a temperature optimum ≥32 °C. With the addition of daily temperature variation, however, both development rate and survival were significantly reduced at 32 °C compared with 30 °C (*P *=* *0.014 and *P *<* *0.0001 for development and survival, respectively). This means that the temperature optimum in a real-world fluctuating environment (what we call the ‘realized’ optimum temperature, r*T*_o_) will be lower than the ‘fundamental’ optimum temperature (*T*_o_) estimated under constant conditions.

**Figure 3:**
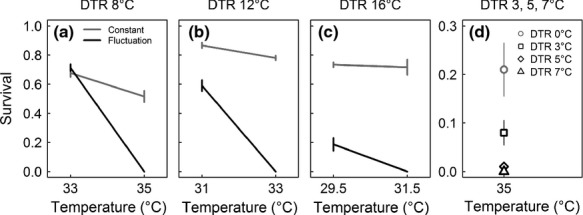
Impact of variable temperatures on the critical maximum temperature. Survival of *Anopheles stephensi* mosquito immatures at various mean temperatures (*x*-axis) and daily temperature ranges (DTRs) of (a) 8 °C, (b) 12 °C, or (c) 16 °C. Note that rCT_max_ is lower than the fundamental CT_max_ of 36 °C in a constant environment (Fig. 2b). (d) Effect of smaller DTRs around a mean temperature of 35 °C. Vertical error bars represent the standard error of the mean.

Similar patterns occurred with the critical maximum temperature, CT_max_ (Fig. 3a–c). At a constant temperature of 35 °C, larvae survive through to adulthood (Fig. 3a and d), indicating a CT_max_ clearly >35 °C. With the addition of a DTR of 8 °C, however, survivorship was reduced to zero indicating a ‘realized’ critical maximum temperature (rCT_max_) <35 °C. With increasing DTRs of 12 and 16 °C, the rCT_max_ values were reduced further to <33 and <31.5 °C, respectively. Varying DTR around a single mean temperature of 35 °C yielded similar results. With a DTR of 7 °C, no mosquitoes survived to adult eclosion. As the extent of the DTR reduced from 5 to 3 to 0 °C, survivorship gradually increased indicating a shift in rCT_max_ (Fig. 3d).

### Theoretical studies

We examined the consequences of daily temperature variation for 29 terrestrial insect species using rate summation (i.e., summing fitness at hourly intervals as temperature fluctuates across the standard fitness curve in line with the average DTR experienced by the insect). In Fig. 1, we present an illustrative example for one temperate species (note fitness is scaled to 1 to enable comparisons of relative fitness). The rate summation approach yields qualitatively similar patterns to our empirical studies, whereby fluctuation at low mean temperatures increases fitness, while fluctuation around high mean temperatures reduces fitness. Fluctuation also reduces the temperature optimum (r*T*_o_ < *T*_o_) (together with the maximum attainable fitness at this optimum), as well as the critical maximum temperature (rCT_max_ < CT_max_). Commensurate with the lower temperature optimum, the TSM is reduced in the fluctuating environment from 16.6 to 13.5 °C.

**Figure 4:**
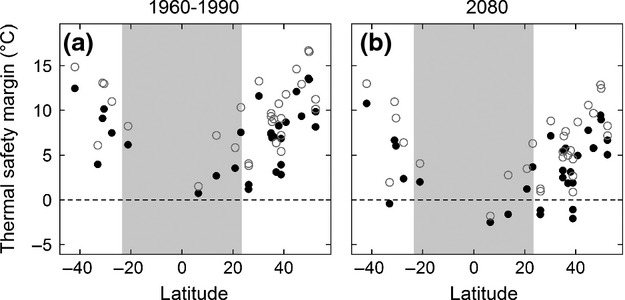
Effects of daily temperature variation and climate warming on thermal safety margins across latitude. Thermal safety margins for 29 terrestrial insect across latitude, as estimated for (a) the period 1960–1990 or (b) the period 2080. Open gray circles represent estimates based on the mean habitat temperature, black solid circles estimates based on the average habitat temperature range. The gray area represents the tropics.

Extending this approach to the full set of species results in reductions in TSMs ranging from 0.8 to 4.5 °C (Fig. 4a). The decrease in TSM applies to all species across latitudes; because daily temperature variation lowers the realized temperature optimum and brings it closer to the prevailing habitat temperature, we predict species to have increased sensitivity to climate warming.

The increased sensitivity is confirmed by substituting the contemporary climate data with projections from the HadCM3 climate scenario for 2080 (considering not only changes in mean temperature but also the forecast changes in temperature variation for species-specific locations, see Figure S1). As expected, with warmer habitat temperatures across the board, the TSMs of all species are reduced. However, with mean temperatures and the fundamental fitness curves, only one of the TSMs was reduced to zero or below, indicating some remaining buffer against negative impacts on fitness for the majority of species. With daily temperature fluctuations and the realized fitness curves, on the other hand, effects for all species were more severe and seven species had negative TSMs (Fig. 4b).

## Discussion

Our empirical and theoretical investigations demonstrate that key elements of thermal sensitivity (*T*_o_, CT_max_, TSM), as well as the overall shape of the thermal reaction norms, depend not only on mean temperatures but also on the extent of daily temperature variation.

The empirical and theoretical effects of temperature variation follow a number of previous studies (Siddiqui *et al*., 1973; Worner, 1992; Liu *et al*., 1995; Mironidis & Savopoulou-Soultani, 2008; Paaijmans *et al*., 2010; Bozinovic *et al*., 2011) and are consistent with Jensen's inequality (Ruel & Ayres, 1999), whereby fluctuation over a concave function (i.e., the cold end of a reaction norm) results in a net increase in a rate process (or fitness), fluctuation over a convex function (i.e., the warmer part of a reaction norm) a net decrease, and fluctuation over a linear function, no net change. There is evidence from a range of taxa that short-term temperature dynamics can influence both the ecology (Rohr & Raffel, 2010; Bozinovic *et al*., 2011; Estay *et al*., 2011; Folguera *et al*., 2011; Hamilton *et al*., 2012; Raffel *et al*., 2013) and evolution (Martin & Huey, 2008; Kingsolver *et al*., 2009; Asbury & Angilletta, 2010) of ectotherm life-history traits (and note that temporal variation in temperature can also affect hibernating endotherms (Boyles & McKechnie, 2010)). The extent to which such effects derive from rate summation alone remains unclear. Additional physiological mechanisms such as production and breakdown of heat shock proteins (McMillan *et al*., 2005) could further exacerbate the influence of temperature variation, especially toward the extremes of thermal reaction norms.

Defining the temperature extremes and critical thermal limits is important for understanding species distribution limits and responses to climate change (Deutsch *et al*., 2008; Huey *et al*., 2009; Santos *et al*., 2011; Piyaphongkul *et al*., 2012; Ribeiro *et al*., 2012). Estimates of CT_max_ are highly sensitive to the methodology used and can vary depending on factors such as heating rates, insect age, and body mass (Bowler & Terblanche, 2008; Santos *et al*., 2011; Ribeiro *et al*., 2012), as well as the specific response parameter studied (Santos *et al*., 2012). The observed reduction in CT_max_ under fluctuating temperature conditions adds another layer of complexity to accurately defining this critical parameter.

Exactly how measures such as TSM influence vulnerability to climate change is unclear. A species' vulnerability to climate change will depend on a range of factors, and unraveling the relative importance of these creates a number of research challenges. For example, we have focused on temperature as a single variable but other abiotic (e.g., rainfall, humidity, CO_2_) and biotic stressors (intra- and interspecific competitors, predators and parasites etc.) can interact with temperature to determine overall fitness (Clusella-Trullas *et al*., 2011; Hamilton *et al*., 2012). In addition, while it is generally true that smaller terrestrial ectotherms conform to ambient temperature (Stevenson, 1985a), certain ectotherms may limit temperature extremes via thermal behavior (Stevenson, 1985b; Huey *et al*., 2012) and hence, modulate the short-term influence of temperature variability. In the longer term, ectotherms can potentially modify responses through genotypic adaptation and/or phenotypic plasticity (Atkins & Travis, 2010; Chevin *et al*., 2010), further altering thermal reaction norms. Moreover, overall fitness is a composite metric and it is important to partition temperature–fitness relationships into component parts, such as fecundity, development, and survivorship (Folguera *et al*., 2011; Amarasekare & Savage, 2012). In our experiments, we focused on two immature life-history traits (development time and survival) to demonstrate the effects of fluctuation on thermal reaction norms, yet overall insect fitness is determined by a suite of traits, including adult longevity and life-time reproductive output, each with potentially different reaction norms (e.g., see Mordecai *et al*., 2013). How multiple reaction norms combine is unclear but given the potential for trade-offs, temperature optima for overall fitness might differ from those of individual traits.

These issues notwithstanding, we follow the argument of Huey *et al*. (2012) that thermal fitness curves provide a convenient, fundamental descriptor of how temperature influences fitness of ectotherms. In this regard, TSM represents a comparative metric to characterize sensitivity (Deutsch *et al*., 2008; Jaramillo *et al*., 2009; Clusella-Trullas *et al*., 2011; Krenek *et al*., 2012). Other proxies of sensitivity, such as warming tolerance, tolerance range, and fitness breadth rely similarly on attributes of the thermal fitness curve (Huey *et al*., 2012).

Climate change can lead to phenological change, species' range shifts and even extinction of plants and animals (Easterling *et al*., 2000). Most studies to date use the standard fitness curves derived from constant temperature experiments combined with mean habitat temperatures to assess such climate-induced risks. Our results indicate that inclusion of daily temperature dynamics can generate substantial shifts in these fitness profiles. The general lowering of TSMs and CT_max_, together with the potential for altered ecological interactions under fluctuating temperature conditions (e.g., Duncan *et al*., 2011; Hamilton *et al*., 2012), might provide some explanation of the observation that terrestrial ectotherms are shifting much faster in response to climate change than previously predicted (Chen *et al*., 2011). To better understand the role of environmental temperature, there is a clear need to integrate ecologically realistic thermal regimes into a wider range of empirical studies (Rohr *et al*., 2011; Niehaus *et al*., 2012), matching thermal regimes with the operative body temperatures experienced by organisms in the field.

## Supporting Information

Figure S1 Mean temperatures and daily temperature ranges, and changes therein as a result of climate warming. (a) mean habitat temperature (T_h_; as recorded in the 1960–1990 period) and daily temperature range (DTR), and (b) the change in mean habitat temperature (T_h_) and DTR as a result of warming. The gray area represents the tropics.
