# Supplementary figures and images for: Temperature variation makes ectotherms more sensitive to climate change
Source: Glob Chang Biol. 2013 May 29;19(8):2373–80. doi: 10.1111/gcb.12240 (PMC3908367; doi:10.1111/gcb.12240)

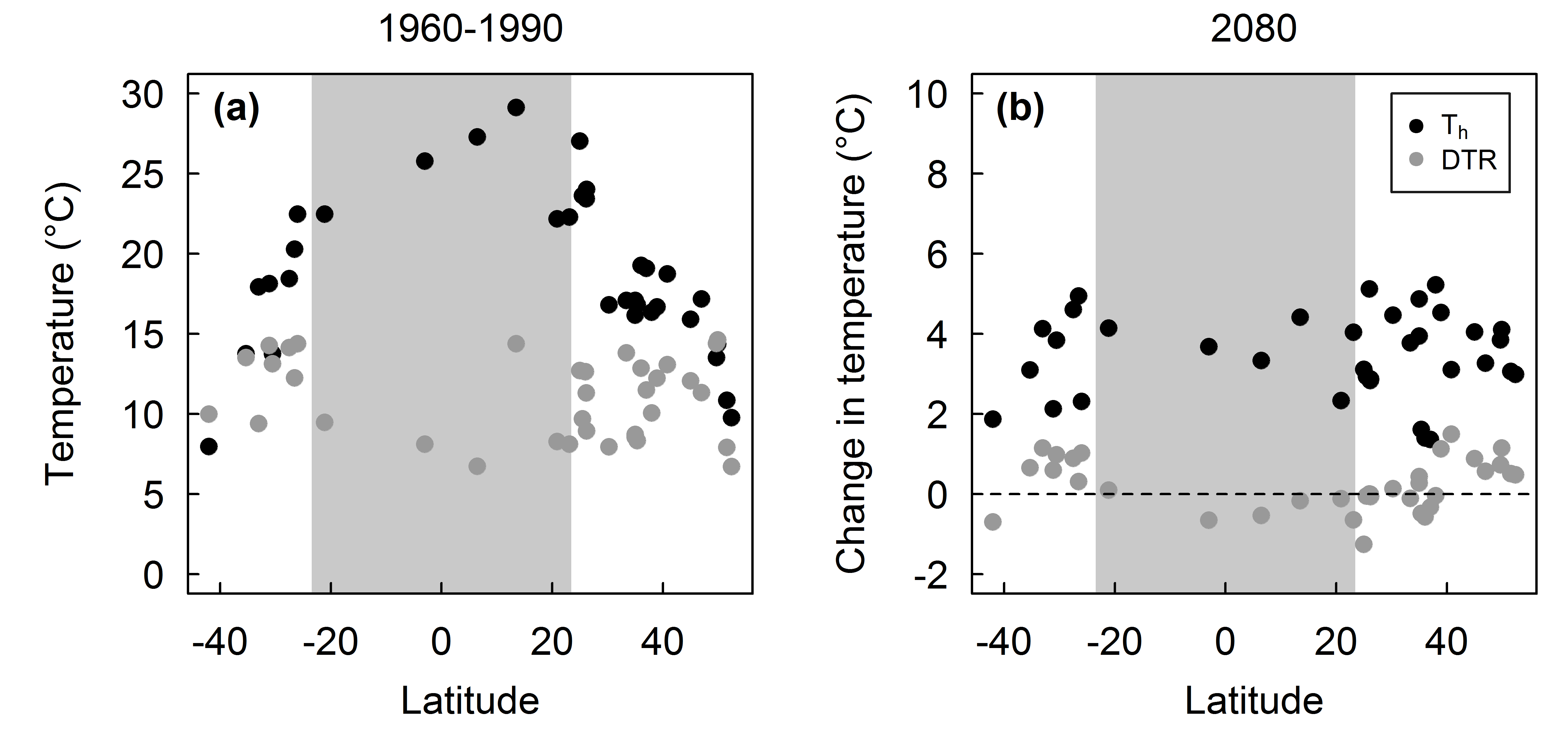

Supplement: Figure S1 — Mean temperatures and daily temperature ranges, and changes therein as a result of climate warming. (a) mean habitat temperature (Th; as recorded in the 1960–1990 period) and daily temperature range (DTR), and (b) the change in mean habitat temperature (Th) and DTR as a result of warming. The gray area represents the tropics. [file gcb0019-2373-sd1.tiff]
